# IoMT-Enabled Computer-Aided Diagnosis of Pulmonary Embolism from Computed Tomography Scans Using Deep Learning

**DOI:** 10.3390/s23031471

**Published:** 2023-01-28

**Authors:** Mudasir Khan, Pir Masoom Shah, Izaz Ahmad Khan, Saif ul Islam, Zahoor Ahmad, Faheem Khan, Youngmoon Lee

**Affiliations:** 1 Department of Computer Science, Bacha Khan University, Charsadda (BKUC), Charsadda 24420, Pakistan; 2 Department of Computer Science, Institute of Space Technology, Islamabad 44000, Pakistan; 3 Department of Computer Engineering, College of Electrical and Mechanical Engineering, National University of Sciences and Technology (NUST), H12, Islamabad 44000, Pakistan; 4 Department of Computer Engineering, Gachon University, Seongnam 13120, Republic of Korea; 5 Department of Robotics, Hanyang University, Ansan-si 15588, Republic of Korea

**Keywords:** pulmonary embolism, computed tomography scans, computer-aided diagnosis (CAD), deep learning, CNN, DenseNet201

## Abstract

The Internet of Medical Things (IoMT) has revolutionized Ambient Assisted Living (AAL) by interconnecting smart medical devices. These devices generate a large amount of data without human intervention. Learning-based sophisticated models are required to extract meaningful information from this massive surge of data. In this context, Deep Neural Network (DNN) has been proven to be a powerful tool for disease detection. Pulmonary Embolism (PE) is considered the leading cause of death disease, with a death toll of 180,000 per year in the US alone. It appears due to a blood clot in pulmonary arteries, which blocks the blood supply to the lungs or a part of the lung. An early diagnosis and treatment of PE could reduce the mortality rate. Doctors and radiologists prefer Computed Tomography (CT) scans as a first-hand tool, which contain 200 to 300 images of a single study for diagnosis. Most of the time, it becomes difficult for a doctor and radiologist to maintain concentration going through all the scans and giving the correct diagnosis, resulting in a misdiagnosis or false diagnosis. Given this, there is a need for an automatic Computer-Aided Diagnosis (CAD) system to assist doctors and radiologists in decision-making. To develop such a system, in this paper, we proposed a deep learning framework based on DenseNet201 to classify PE into nine classes in CT scans. We utilized DenseNet201 as a feature extractor and customized fully connected decision-making layers. The model was trained on the Radiological Society of North America (RSNA)-Pulmonary Embolism Detection Challenge (2020) Kaggle dataset and achieved promising results of 88%, 88%, 89%, and 90% in terms of the accuracy, sensitivity, specificity, and Area Under the Curve (AUC), respectively.

## 1. Introduction

The Internet of Things (IoT) has transformed the landscape of the Internet [1,2,3]. It has enabled the automation and integration of real-world objects to enact smart services in various domains such as city planning and management, transportation systems, homes and buildings, and healthcare systems [4,5,6]. Given this, healthcare has always remained at the forefront of technology adoption. However, smart healthcare systems face numerous challenges while enabling IoMT-based solutions, such as pre-processing the generated data, computational power for training an accurate model, and designing a suitable model.

With an estimated death toll of 180,000 per year caused by Pulmonary Embolism Disease (PED) in the US alone, PE is considered a deadly disease [7]. It is caused by a blood clot in the lung vessel that blocks the blood supply to the lung or a part of the lungs. The clot can be formed anywhere in the body, and further travels to the lungs [8]. This disease has a higher mortality rate than lung cancer [8]. Usually, patients with this disease can witness low oxygen saturation, difficulties in breathing, chest pain, and low blood pressure [9]. Besides these symptoms, the patients may also suffer from ischemia or right-sided heart failure [10].

An early diagnosis of this disease can reduce the mortality rate. Previous studies show that the mortality rate is decreased to 2% when diagnosed earlier and provided proper treatment [11]. For diagnosis, doctors, and radiologists primarily use CT scans in clinical practices. Typically, each CT comprises 300–500 slices, making it difficult for a radiologist to go through them and examine each pulmonary artery. Further, this process is time-consuming and prone to error [10]. Therefore, there is a need for an automatic CAD system.

On the other hand, deep learning has shown incredible performance in computer vision and natural language processing. Deep Convolutional Neural Networks (DCNNs) are the game-changer tool in the domain. They have solved many problems, including machine translation, image segmentation, image classification, speech, and facial recognition tasks. Further, these techniques are also known for obtaining higher accuracy than humans. DCNNs are also used for medical diagnosis problems inspired by such performances. Rajpurkar et al., in 2019 [12], applied DenseNet121 architecture, which was named chexnet, and achieved a human-level accuracy. Similarly, such CNN models are used for Parkinson’s detection [13], brain tumor segmentation [4], COVID-19 detection [14,15], and ischemic stroke lesion segmentation [16]. The performance of DCNNs in medical diagnosis convinced the authors to apply DCNNs on CT scans to detect PE better and reduce the error rate.

Previously, many CAD systems were proposed, such as [17,18,19], in order to assist radiologists and speed up the diagnosis process. However, these systems have highly false markings when reviewed by expert radiologists [11]. Even though CNNs have achieved high accuracies in many domains, these networks mainly suffer from a series of problems, such as gradient vanishing, a non-availability of enough training data, and overfitting, that remain to be solved. Such problems have restricted the performance of the CNN model. In our scenario, the main problem is gradient vanishing during the training in deeper CNNs. The value of the gradient gets smaller and smaller. Thus, the weights do not update and restrict the neural network from learning. Solutions to this problem are somehow addressed in DenseNet architecture, where each layer is connected to the previous layers directly [20]. These direct connections among the layers allow the network to overcome gradient vanishing. We utilized DenseNet201-layer deeper architecture for PE classification into nine classes, positively affecting the results and reducing the error rate by 3%.

The major contributions of this paper are given below:

1. The paper presents a deep-learning-based PE detection CAD system.
2. The proposed method performs feature extraction using DenseNet201 with customized, fully connected decision-making layers.
3. The paper also contributes to overcoming the gradient vanishing problem in CNNs for PE detection.
4. The customized model was trained on the Radiological Society of North America (RSNA)-Pulmonary Embolism Detection Challenge (2020) Kaggle dataset and showed promising results of 88%, 88%, 89%, and 90% in terms of the accuracy, sensitivity, specificity, and AUC.
5. The proposed system reduced the error rate by 3% of CAD in PE detection.

The organization of this article is as follows: Section 1 introduces the paper. Section 2 presents state-of-the-art relevant work. The Materials and Methods are presented in Section 3. Subsequently, results are discussed in detail in Section 4, and Section 5 concludes the paper with future directions.

## 2. Related Work

This section discusses relevant work on using deep learning models to predict PE. Further, an overview of related work is shown in Table 1.

Tajbakhsh et al. [21] used a CNN to detect PE from Computed Tomography Pulmonary Angiogram (CTPA). They used 121 CTPA datasets with 326 individual emboli. The proposed approach outperforms traditional machine learning techniques by detecting individual emboli with an 83% sensitivity at two false positives per scan and a 71% sensitivity with the same number of false positives. The authors showed a CNN-based strategy.

To detect PE in CT scans, a two-stage CNN was used by Xing Yang et al. [10]. A 3D fully CNN retrieves 3D feature hierarchies effectively to propose a candidate from original CT scans in the first phase. The ResNet-18 classifier was utilized in the second step to eliminate false positives using vessel-aligned candidate transformation and 2D classification. Experiments were conducted on two datasets: a public dataset from the PE challenge with 20 patients for training and another 20 for testing, and their personal dataset with 129 cases. Ninety-nine patients were gathered from local hospitals, with the other 30 patients coming from a public database. On the public dataset, the authors achieved a sensitivity of 75.4% with two false positives per scan and a localization error of 0 mm, and  on the other dataset, they achieved a sensitivity of 76.3 percent with two false positives per scan and a localization error of 0 mm, respectively.

Weifang Liu et al. [22] trained a CNN model to detect and compute acute PE (APE) clotting lesions from CT scans. The U-Net architecture based on a deep learning CNN was used. The author divided the complete CTPA into two portions. A total of 590 studies were trained for clot segmentation (460 with APE and 130 without APE). The validation set comprised 288 studies, including 186 studies with APE and 102 studies without APE, to detect and calculate the clot volume. The U-Net results showed 94.6 and 76.5 in terms of sensitivity and specificity, with an AUC of 0.926, respectively. In contrast, DL-CNN has a high AUC for identifying PE and can measure the clot burden of APE patients, decreasing the clinical strain.

A PENet model was utilized by Huang et al. [7] to detect PE. PENet is a 3D CNN model comprising 77 layers. The model was trained on the kinetics-600 dataset before being fine-tuned on the CTPA dataset obtained from an academic institution. The performance was examined in the diagnosis of PE. Data was acquired from two other institutions, i.e., one from the same university (Stanford), and the other from a different university (Intermountain), in order to evaluate the generalizability of the model in the context of independent population datasets. On the internal test set, the authors achieved an Area Under the Receiver Operating Characteristics (AUROC) of 0.84, whereas, on an external dataset, the AUROC was 0.85. Similarly, on the internal dataset, they achieved 0.82, 0.73, and 0.77 in terms of specificity, sensitivity, and accuracy. Further, they achieved 0.80, 0.75, and 0.78 in terms of specificity, sensitivity, and accuracy, respectively, on the external dataset. The PENet model also outperformed ResNET3D, ResNetXt3D, and DenseNet3D. The research shows that PENet outperformed the current medical field and health care.

Weikert et al. [23] utilized an AI-powered algorithm to auto-detect PE. Training of the proposed technique is based on Resnet architecture. A region recommendation stage and a false positive stage are the two steps involved in their model. The authors employed a substantial training dataset of 28,000 CTPAs in this study. The proposed method obtained a 92.7% sensitivity and a 95.5% specificity. The AI prototype algorithm shows the high performance of the diagnostic accuracy in detecting PE. The authors discovered that evaluations with central emboli had a maximum recognition rate (95%), followed by examinations with segmental emboli (93.3%). The detection rate for sub-segmentally situated emboli was the lowest, at 85%.

Aditya Mohan et al. [24] devised a deep learning model based on CNN for detecting and classifying PE. They used the CTPA dataset from the Radiological Society of North America (RSNA). The authors used an Xception CNN for feature extraction and then used these features for classification by transfer learning. The Xception CNN model achieved an accuracy above 90% on the validation test set.

A two-stage detection pipeline using 3D images was developed by Rajan et al. [25]. The suggested method is a generalizable two-stage diagnostic pipeline for PE. In the first stage, to achieve segmentation, U-Net architecture was used. In the second stage, a convolutional-LSTM based classifier was used via Machine Instance Learning (MIL) stage to detect PE. They used a CT dataset consisting of 1874 positive and 718 negative data. They achieved an AUC of 0.94 on the validation test and 0.85 on the test set. The offered method achieved a significant result. This study can be used for planning and designing an effective pipeline.

Tuomas Vainio et al. [26] proposed a deep learning CNN model to identify hypo-perfusion in chronic PE (CPE) from CTPA. A 12-layer-deep U-Net type CNN was trained. CTPA was employed on 25 patients with CPE and 25 patients without PE. The data was further divided into training, testing, and validation: 48%, 12%, and 40%, respectively. The predicate segmentation of this model showed an AUC of 0.87. The work demonstrates the effectiveness of a deep learning method for detecting hypoperfusion in CPE on CTPA. The findings suggest that CNNs can help clinicians to diagnose and treat patients with CPE by automatically assisting them.

Tuomas Vainio et al. [26] were the first to declare a deep learning CNN method to identify shock CPE. The predicate segmentation of this model showed an AUC of 0.87. The deep learning system outperformed the CTPA in diagnosing hypoperfusion in CPE in this study. The findings support the use of CNNs in diagnosing and managing patients with CPE.

Nahid ul Islam et al. [8] presented a study of deep learning for diagnosing PE. The authors used the RSNA PE CT dataset. They presented two levels of analysis. The authors compared self-supervised learning against supervised learning and contrasted CNNs against vision transformers at the image level. The exam level primarily focused on comparing conventional classifications with multiple instance learning. These efforts are beneficial in three ways: (1) a detailed evaluation of various deep learning approaches for PE detection; (2) a comparison of architectural model initialization and learning approaches on a large scale; (3) a comparison to the current performance standards, where the authors used different architectures, SeResNextSe, ResNext50, SeXception, DenseNet121, ResNet18, and ResNet50, which achieved an AUC of 0.88, 0.89, 0.88, 0.88, 0.87, and 0.86, respectively.

For the prediction of PE, a two-stage attention-based CNN—long short-term memory (CNN-LSTM) network was developed by Sudhir Suman et al. [27]. A CNN and the LSTM+Dense sequence model make up the two-stage attention-based model. CNN was employed to take possession of the image properties and examine labels. To give network information for each slice, the sequence stage combines these features with the acquired long-range dependencies. On the test set, the CNN-LSTM network had an AUC of 0.95 for detecting the existence of PE. The model was trained using the N = 7279 studies in the RSNA-STR PE CT dataset and was tested on a curated dataset of 106 studies in-house.

Highly generalizable deep learning algorithms, named Emboleye, were developed for detecting PE by Ryan Schmid et al. [28]. They used a large and diverse dataset, including 305,074 CTs collected from 2000 hospitals in the U.S. The method was established and tested with angiography in mind. During an angiography examination, Emboleye had an AUC of 0.79 and a specificity of 0.99. Regarding non-angiography, the algorithm had an AUROC of 0.77 and a specificity of 0.99. In comparison to prior recent work on detecting PE, the authors obtained a significant performance in this research.

## 3. Materials and Methods

### 3.1. Data Set

**Figure 1:**
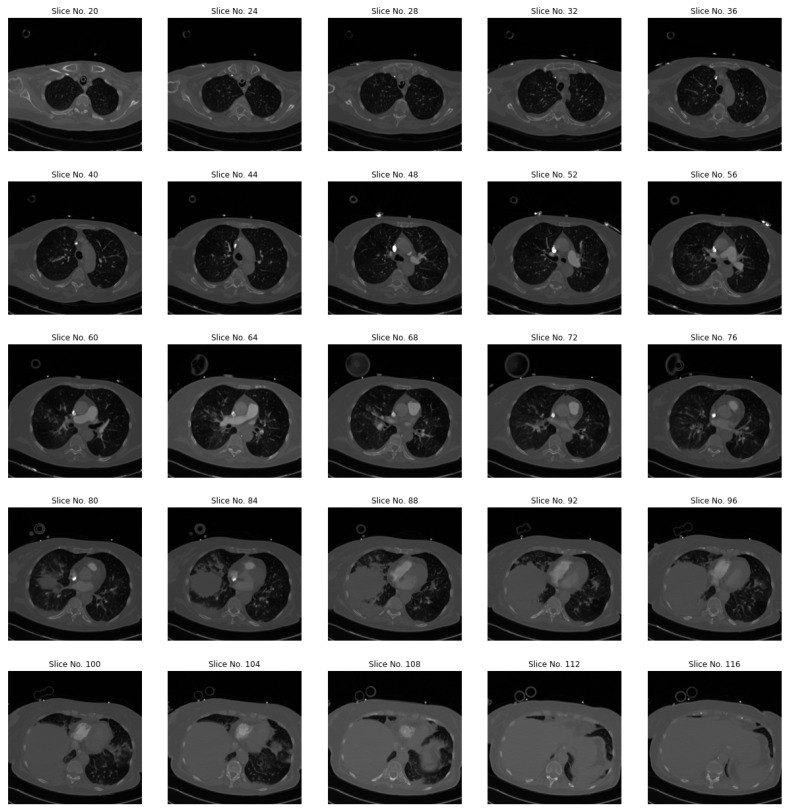
Sample CT images of a randomly chosen subject from the dataset.

The data used in this study was obtained from the Kaggle repository [29]. This dataset is the composition of three different datasets that RSNA previously released, with the contribution of five other countries and institutes, including Unity Health, Toronto Canada, Federal University Brazil, Alfred Health, Australia, KOC University, Turkey, and Stanford University California. This makes it a larger annotated dataset with a rich diversity of samples around the globe. Moreover, it contains soft-tissue images of chest CT scans with a thickness of 2.5 to 3.0 mm, followed by pulmonary angiography protocols [30]. Figure 1 presents CT scans of a randomly chosen study of the dataset, starting from slice no. 20 to 116, with four skips.

#### 3.1.1. Annotation Process

The annotation of the dataset was a joint effort between the RSNA and the Society of Thoracic Radiology (STR), who invited different volunteers (field experts) to serve as expert annotators for the dataset. Every volunteer went through a detailed evaluation. They were given the task of providing a diagnosis of 20 test cases. A total of 86 volunteers obtained a score of more than 65%, which makes the ground to invite them for annotation of the dataset [30].

#### 3.1.2. The Final Dataset

The whole dataset is composed of 2,995,147 CT images belonging to 12,195 subjects. They are further divided into training and testing sets of 77% (2,305,802 images) and 23% (686,345). However, the size of the dataset is decreased by 30% due to the space constraint on Kaggle. In both the training and testing sets, PE-negative samples are excluded randomly [30]. The dataset available on Kaggle comprises 2,322,685 images over 9446 examinations, where 1,790,594 images over 7279 examinations belong to the training set and 532,091 images over 2167 examinations belong to the test set. Since the test set available on Kaggle lacks labels, we choose 10 percent of samples of each class, making it a particular test set for evaluating our model’s performance. We keep the specific test set utterly separate from the training set (i.e., none of these images are included in the training).

### 3.2. Slices Selection and Pre-Processing

We uniformly exclude the first 10 slices from all the training and testing sets. Similarly, the last 15 slices of each study are also excluded from training and testing. The main reason for the exclusion is to reduce computational complexity. Most of these slices do not carry enough information related to PE compared to the middle-order slices. CTs commonly suffer from noise that is produced by medical imaging equipment. We apply a uniform strategy for all the classes (i.e., preprocessing steps) to reduce the noise ratio, including contrast stretching, histogram equalization, and adaptive equalization. Therefore, we needed to adjust the Window Level (WL) and Window Width (WW) settings of CTs. Adjusting the WL and WW of a CT image is used to optimize the visibility of the structures of interest within the image. The WL refers to the level of the grayscale at which the center of the window is set, whereas the WW represents the range of grayscale values displayed within the window. A smaller WW typically results in a high-contrast image, whereas a larger WW will produce a lower-contrast image. The appropriate WL and WW values can depend on the structures being visualized and their contrast within the image. In the case of PE, a higher contrast setting is helpful for better visualization of small blood clots in the pulmonary arteries. It is generally recommended [31,32,33,34] to use the smallest WW that allows for the necessary structures to be seen, as it produces the highest-contrast image.

The Algorithm 1 adjusts the WL and WW settings for high contrast in CT scans. The input into the algorithm is the CT image (denoted as $I_{CT}$), and the maximum allowed values for WL and WW (denoted as $WL_{max}$ and $WW_{max}$). The procedure first initializes the WL and WW values to the minimum and maximum intensity values in the CT image, respectively. It then enters two while loops, where, in each iteration, the WW value is decremented by one until it is less than or equal to $WW_{max}$, and the WL value is incremented by one until $WL+WW\leq WL_{max}$. Once both while loops have been completed, the final WL and WW values are returned.
**Algorithm 1** Adjusting WL and WW for high-contrast setting in CT scans.**procedure**AdjustWLWW($I_{CT},WL_{max},WW_{max}$)    $WL\leftarrow \mathrm{minimum}\;\mathrm{intensity}\;\mathrm{value}\;\mathrm{of}\;I_{CT}$    $WW\leftarrow \mathrm{maximum}\;\mathrm{intensity}\;\mathrm{value}\;\mathrm{of}\;I_{CT}-WL$    **while** $WW>WW_{max}$ **do**        WW←WW−1    **end while**    **while** $WL+WW>WL_{max}$ **do**        WL←WL+1    **end while**    **return** WL,WW**end procedure**

### 3.3. Proposed Framework

**Figure 2:**
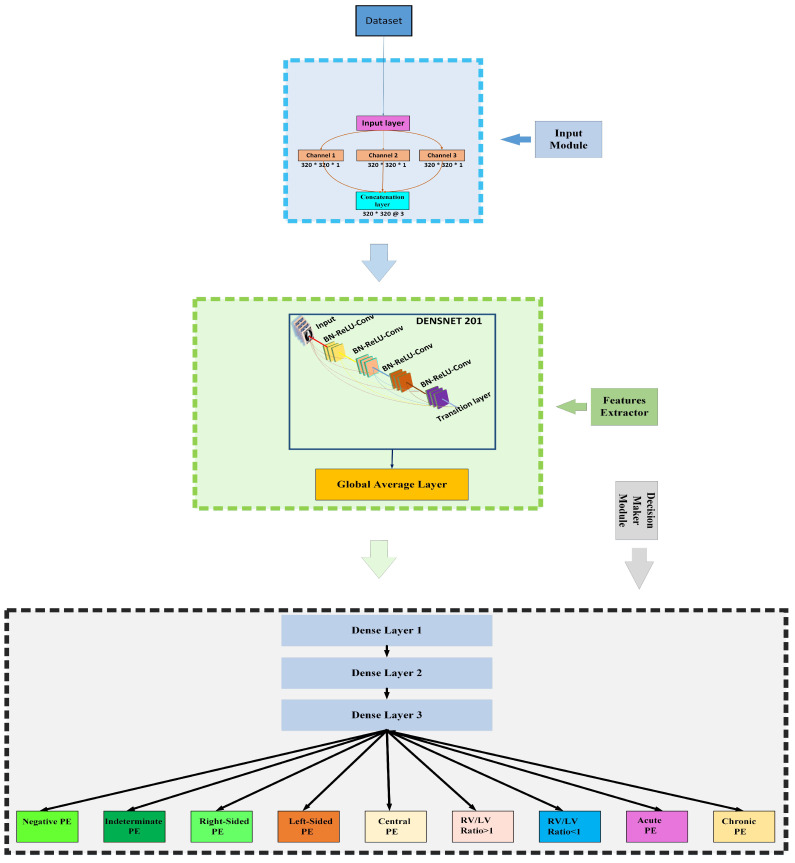
Proposed framework (RV = right ventricle, LV = left ventricle).

Our proposed framework for PE detection can be divided into three modules: the first one is the input module, the second one is the DenseNet201-based feature extractor, and the last one is the decision-making module, Figure 2 illustrates our proposed framework. All of these modules are discussed in detail in the following sections.

#### 3.3.1. Input Module

The input module obtains the CT images effectively and then forwards them to the feature extractor module. In this module, we used three parallel layers, where each layer handles a separate image channel. Each layer takes an input image in the dimension of 320×320×1 and forwards it to the next layer. Then, the output of all these layers is concatenated in the shape of 320×320×3 on the last layer of the input module, which is then forwarded to the feature extractor module.

#### 3.3.2. Towards DenseNet

In the computer vision domain, DenseNet architecture is known for its record-shattering performance in object recognition tasks, such as CIFAR-100 [35] and ImageNet [36] competitions. However, the spike in developing deep learning models started in 2012 when Alexnet, a novel CNN architecture, outperformed in a large-scale image localization task and secured the first position. Similarly, this architecture also secured second place in the classification task [37]. The design of Alexnet consisted of only eight layers, where five were convolutional and three were fully connected. It was a simple CNN model compared with recent state-of-the-art architectures; however, its encouraging results have attracted the focus of AI researchers toward deep learning. We have witnessed advanced CNN models with higher performance rates in the current era. However, these models suffer from gradient vanishing problems despite achieving excellent results. Gradient vanishing is a problem in the neural networks during the training phase. Eventually, when the model’s training depends on gradient-based learning, the value of the gradient becomes smaller and smaller after alterations, which further restricts the weights from getting updated. Adding shortcut connections in the new CNN models is an effective way to cope with issues such as vanishing gradients. In an attempt to solve the exploding/vanishing gradients when the networks go deeper, the authors in [38] came up with a solution of an improved inception module that comprises shortcut connections and a few deeper branches. For training, highway networks were developed to allow information generated by previous layers to pass to following layers without losing data, easing the training process of deep networks based on the gradient. Moreover, to facilitate the training process and take advantage of wide and deeper CNNs, the DenseNet architecture was proposed. This architecture works on the principle of reusing the feature maps technique. The input to the subsequent layer is the concatenation of all the feature maps produced by all the proceeding layers. The concatenation of the feature maps is explained with mathematical support in Equation (1).

(1) $$z^{l}=H_{1}\left(\left[z^{0},z^{1},\ldots \ldots ,z^{l-1}\right]\right)$$

In this equation, *H* refers to a composite function or non-learning transformation consisting of batch normalization followed by a rectified linear unit function and a convolutional of 3. Moreover, $\left[z^{0},z^{1},\ldots \ldots ,z^{l-1}\right]$ depicts the continuation of all the feature maps. For down-sampling, four dense blocks are introduced. Each one is separated from the other dense block using batch normalization followed by 1×1 convolutional and 2×2 average pooling layers. Compared to the traditional convolutional neural networks, the size (height and width (HXW)) of feature maps are not reduced after the convolutional operation but are halved.

DenseNet201 performs well even with a slower growth rate because its architecture considers feature maps of the network’s global state. As a result, each subsequent layer obtains access to all feature maps from the previous layers [20]. Each layer adds *k* feature maps to the global state.

**Figure 3:**
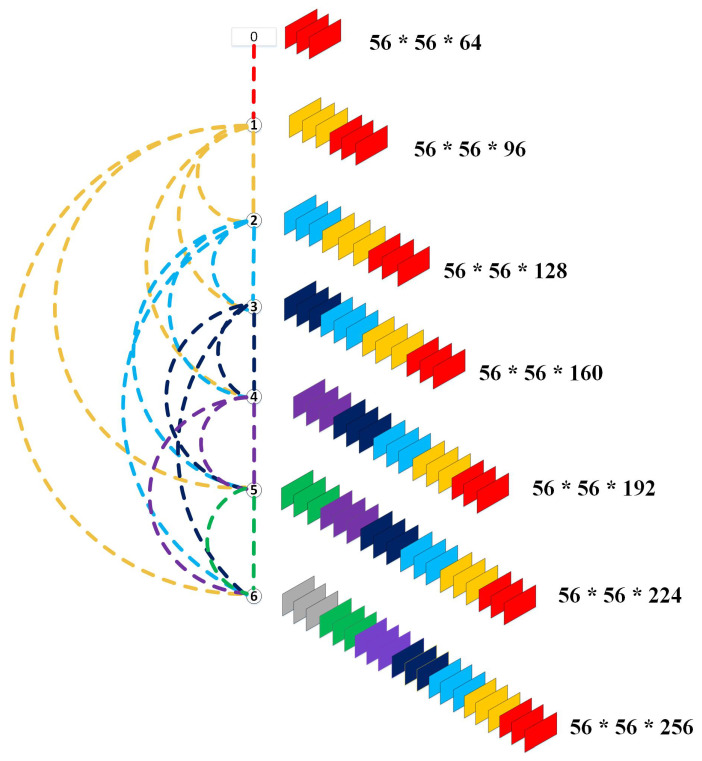
Illustration of feature map concatenation in each layer.

When the network becomes more profound, the number of concatenated feature maps also grows. This growth rate must be restricted. Otherwise, the network would have a high number of parameters. In an attempt to regulate the progression of these feature maps, the growth rate K was designed. In this way, the number of feature maps at the *l*th layer can be calculated as k0+(l−1)×k. In the equation, k0 refers to the total number of channels in the input layer. Figure 3 illustrates the concatenation of feature maps in each layer of the first dense block of DenseNet201, where the value of *k* is 32. Moreover, bottleneck layers were introduced to reduce the computational complexity, with a 1×1 convolutional layer before every 3×3 convolutional layer. We used DenseNet201-layered architecture as a feature extractor. We started the training of DesnseNet201 using its ImageNet weights along with the same structure of convolutional layers as done in [20]. The illustration of our proposed framework can be seen in Figure 2. This module receives input images in the shape of 320×320×3 while producing the output in the form of 1×1×1920, which is then forwarded to the decision-making module.

#### 3.3.3. Decision-Making Module

The section discusses the fully connected layers of our proposed framework, which can be further divided into two-stage layers: intermediate dense layers and classification layers. In the intermediate layers, we used three dense layers with output neurons of 1024, 256, and 64 activated by ReLU functions, whereas, in classification layers, we placed nine dense layers, which are all parallel connected to the last dense layer of intermediate dense layers. Each layer represents its class, with one output node activated by the sigmoid function.

### 3.4. Experimental Setup

We continued our model training for 2 h with the following parameter setup and a batch size of 1000. The dropout ratio was set to 0.5. We used RMSprop as an optimizer, introduced by Hinton [39], which extends the traditional stochastic gradient descent algorithm that utilizes a moving average of the squared gradient to scale the learning rate. This allows RMSprop to adapt the learning rate on a per-parameter basis, making it well-suited for handling complex, high-dimensional data. Additionally, RMSprop has been shown to converge faster and perform better on noisy and non-stationary data. Overall, the use of RMSprop in the classification of PE in CT scans allowed for the efficient and effective training of the deep neural network, ultimately leading to improved performance. We used batch normalization, which normalized the activations of the layers in the network across a batch of input data, which facilitated faster learning and better generalization of the test data. Moreover, we also adopted the early stopping strategy, which prevented over-fitting by monitoring the model’s performance on a validation set and stopping the training process when the performance started to degrade [40]. For all the experiments, we used a Kaggle notebook. Moreover, the Keras library with TensorFlow backend was used for model implementation. Pydicom library was used to handle the CT images in Dicom formats, and the hidden library was utilized in the entire project.

## 4. Results and Discussions

To evaluate the performance of the proposed model for each class on the test set, we use the accuracy, sensitivity, specificity, and AUC as evaluation measures. Typically, the accuracy matrix is used when all the classes are equally important. It can be derived as the number of all correctly classified classes divided by the total number of all the classes, and is mathematically presented in Equation (2), where TP and FN show the true positive and false negative, respectively.

(2) $$A_{ccuracy}=\frac{TP+TN}{TP+TN+FP+FN}$$

However, only the accuracy is not a complete, dependable evaluation measure. In most cases, accuracy does not give a perfect classifier evaluation, especially when the test set is imbalanced. Therefore, the classifier uses sensitivity or recall to define the proportion of actual true positive instances classified as true positive. Equation (3) represents the mathematical model of the sensitivity matrix.

(3) $$S_{ensitivity}=\frac{TP}{TP+FN}$$

On the other hand, specificity is used to evaluate the classifier’s performance towards the true negative: the proportion of true negative and those classified as true-negative. The lower specificity rate indicates that the classifier suffers from a high false negative rate. Mathematically, it can be defined in Equation (4).

(4) $$S_{pecificity}=\frac{TN}{TN+FP}$$

**Figure 4:**
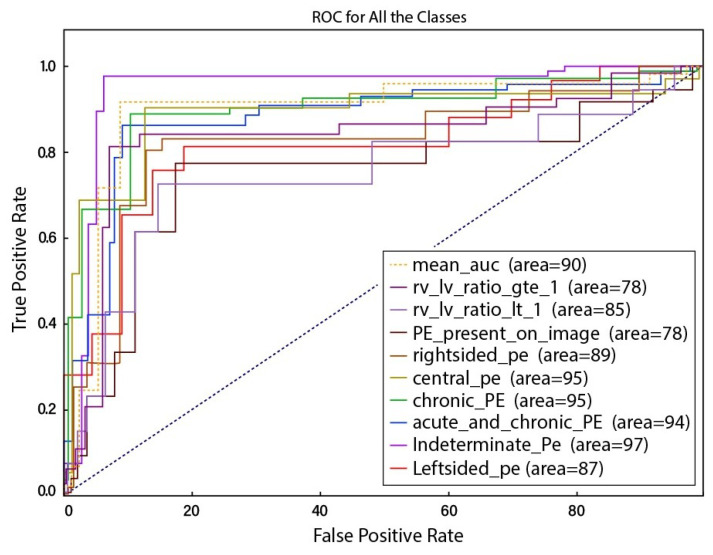
ROC curve for all of the classes.

**Figure 5:**
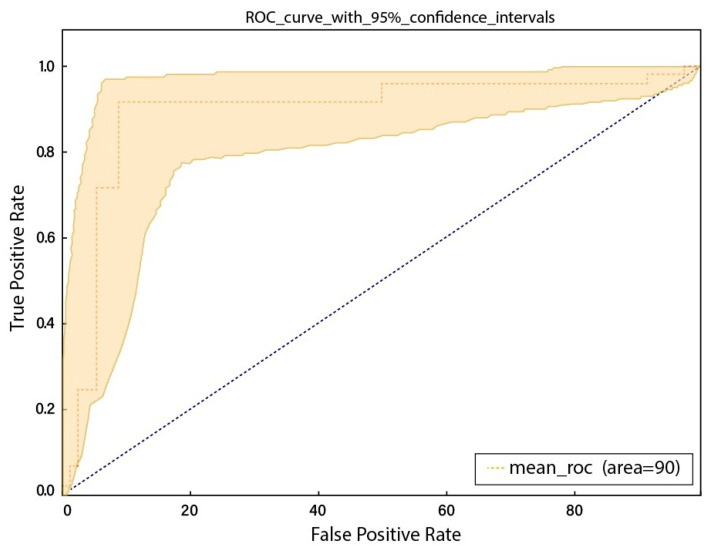
ROC curve with 95% confidence intervals.

To calculate the true positive and false positive rate of prediction algorithms, the Receiver Operator Characteristic (ROC) curve, also known as AUC, is considered as the first-hand measure. Figure 4 represents the ROC curve on the test set. The ROC curve figure presents ten lines, each corresponding to a particular class and indicating the model’s true positive rate (sensitivity) at various classification thresholds. These classes include “RV-LV ratio greater than 1”, “RV-LV ratio less than 1”, “PE present on an image”, “Right-sided PE”, “Central PE”, “Chronic PE”, “Acute and chronic PE”, “Indeterminate PE”, and “Left-sided PE”. The false positive rate (specificity) is plotted on the x-axis, whereas the true positive rate (sensitivity) is plotted on the y-axis. AUC is a metric that summarizes the performance of a classifier across all classification thresholds, with a higher value signifying a better performance. The minimum AUC value among the classes is around 0.78, and this value is achieved by the “RV-LV ratio greater than 1” and “PE present on an image”. The maximum AUC value is around 0.97, and this value is achieved by the “Indeterminate PE” class. The figure also includes a mean ROC curve, which is the average of the individual ROC curves for each class and can compare the model’s overall performance. From Figure 4, it appears that the model has a convincing performance, with an AUC of around 0.9 and lines that are close to the top-left corner of the plot, indicating a high true positive rate and a low false positive rate. Similarly, Figure 5 represents the ROC cure with 95% confidence intervals. Both curves are relatively smooth, indicating that the model can make consistent predictions across a range of classification thresholds.

For all the classes, we calculate the accuracy and error rate separately. Further, we take the mean of all the accuracies as our final accuracy. In all figures, the orange line represents the validation accuracy and the blue line refers to the training accuracy. The y-axis shows the accuracy and the x-axis represents the number of epochs.

**Figure 6:**
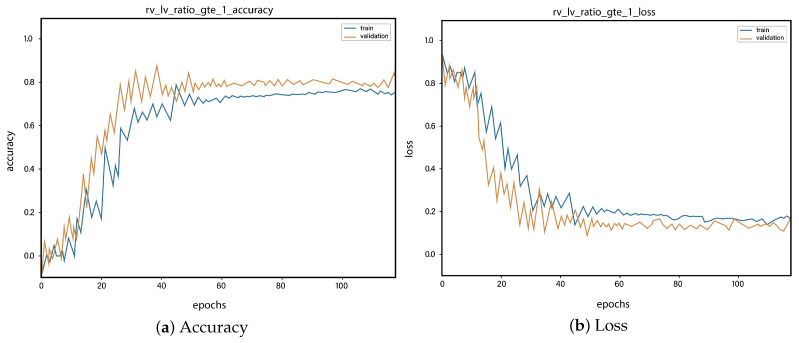
Training vs. validation accuracy and loss for the class: Right-ventricle/left-ventricle ratio greater than 1 PE.

**Figure 7:**
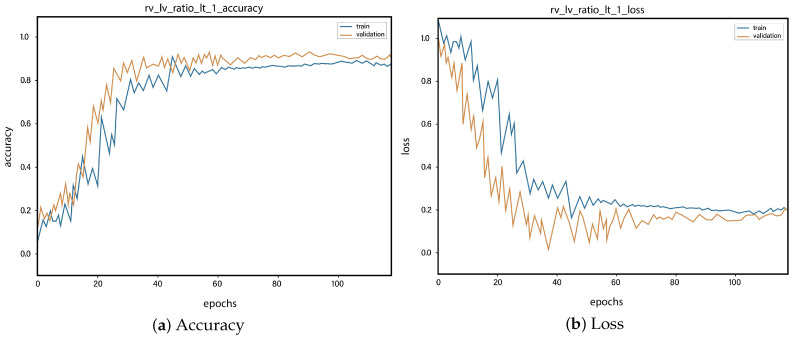
Training vs. validation accuracy and loss for the class: Right-ventricle/left-ventricle ratio less than 1 PE.

**Figure 8:**
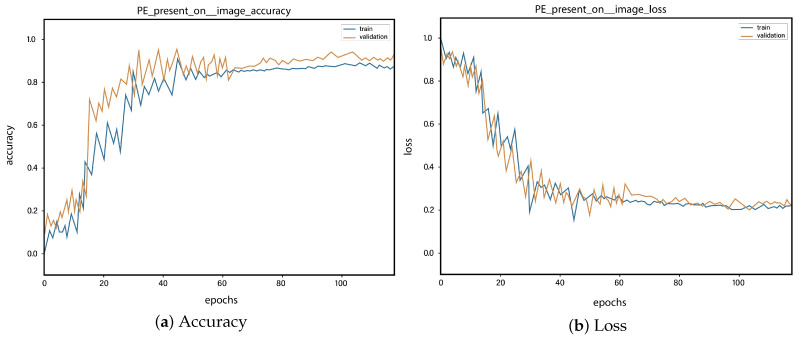
Training vs. validation accuracy and loss for the class: PE present on the image.

**Figure 9:**
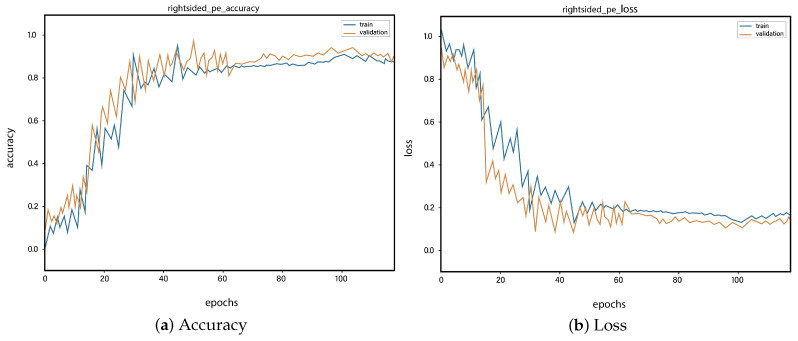
Training vs. validation accuracy and loss for the class: Right-sided PE.

**Figure 10:**
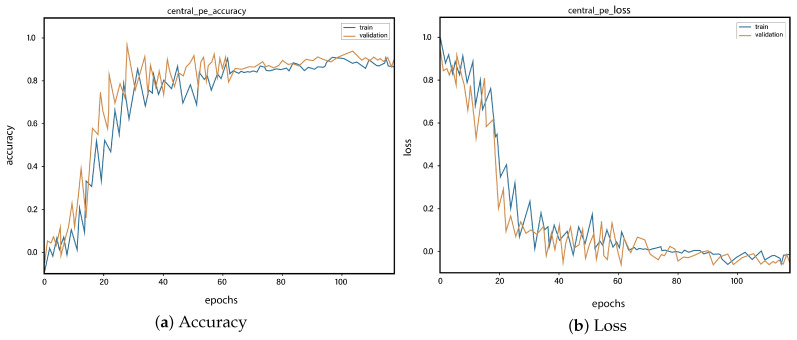
Training vs. validation accuracy and loss for the class: Central PE.

**Figure 11:**
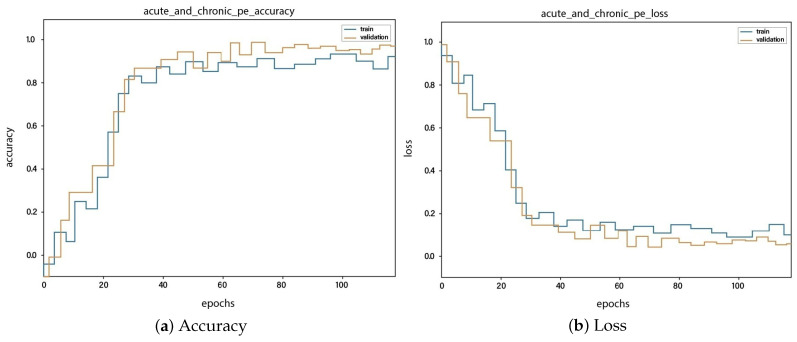
Training vs. validation accuracy and loss for the class: Acute and chronic PE.

**Figure 12:**
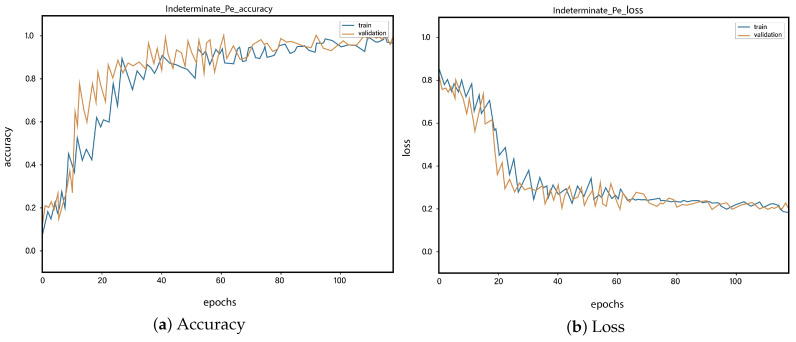
Training vs. validation accuracy and loss for the class: Indeterminate PE.

**Figure 13:**
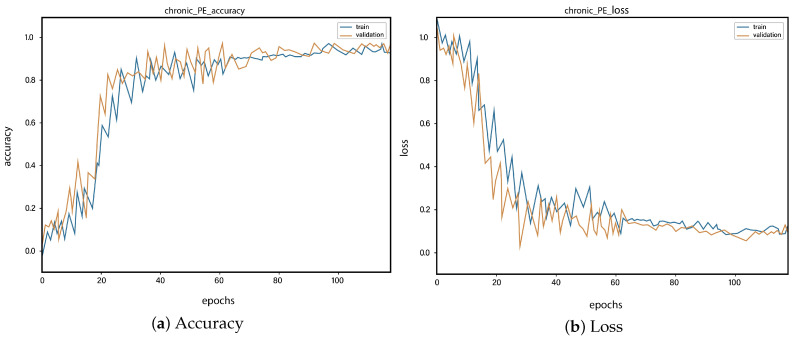
Training vs. validation accuracy and loss for the class: Chronic PE.

**Figure 14:**
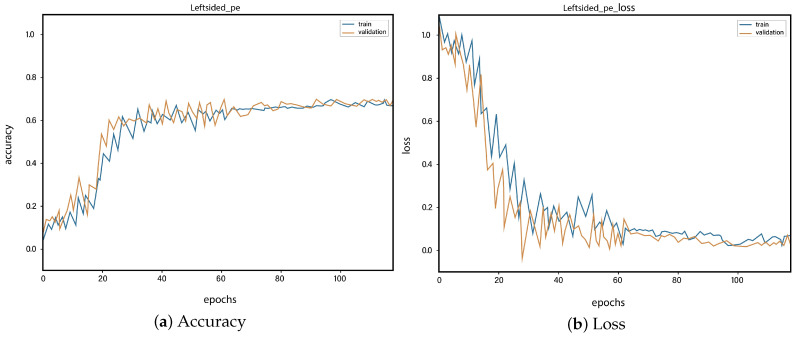
Training vs. validation accuracy and loss for the class: Left-sided PE.

Figure 6a depicts the training and validation accuracy concerning the epochs for the right-ventricle/left-ventricle ratio greater than 1 PE class. Both of the epochs’ accuracies (training and validation) are recorded at around 78%. It is observed that the accuracies fluctuate after several epochs. It is mainly due to the high batch size and the diversities among the samples in the batch size. Figure 6b represents the loss of right-ventricle/left-ventricle ratio greater than 1 PE class. For the right-ventricle/left-ventricle ratio less than 1 PE class, the training and validation accuracy are shown in Figure 7a. Since we have the same batch size for all the classes, the variation in the accuracy (in both training and validation) regarding the epochs is also observed in the class. It can be seen that the recorded score stands at around 85% in terms of accuracy. Figure 7b showed the loss of the right-ventricle/left-ventricle ratio of less than 1 PE class. Similarly, the accuracy of PE present on the image class is shown in Figure 8a, where the accuracy is nearly 78%, and the loss of PE present on an image is shown in Figure 8b. Moreover, Figure 9a shows the accuracy of the right-sided PE class, where the accuracy is nearly 89%, which is nearly 90%. Similarly, Figure 9b represents the loss of the right-sided PE class. In the same way, Figure 10a shows the accuracy of the central PE class. It is observed that the accuracy reached nearly 95% on the validation set. The loss of this class is presented in Figure 10b. For the APE and CPE classes, the training and validation accuracy is shown in Figure 11a. where the accuracy is over 94%. Similarly, the loss of APE and CPE can be seen in Figure 11b. Furthermore, Figure 12a demonstrates the accuracy for the indeterminate PE class, where the accuracy reached 97%. Additionally, Figure 12b represents the loss of the indeterminate PE class. For the CPE class, the accuracy is shown in Figure 13a. It can be seen that the recorded score stands at around 95% in terms of accuracy; in parallel, Figure 13b represents the loss of the CPE class. Figure 14a illustrates the accuracy of the left-sided PE class, where the accuracy is recorded at around 87%. The loss of the CPE class is shown in Figure 14b.

The overall test score can be seen in Table 2 in terms of accuracy, sensitivity, specificity, and AUC. Table 3 shows the summary of previous experiments, in which, our model has shown comparable results.

These results are increased by up to 3% from the state-of-the-art. This improvement in results is mainly caused by addressing the issue of the weight vanishing of CNNs by utilizing DenseNet201.

## 5. Conclusions

To summarize our contributions in this paper, a DenseNet201-based CNN model is proposed to detect PE in nine classes. The experiments are carried out on the RSNA-Kaggle dataset. We exclude the first ten slices and the last ten from each CT scan in the preprocessing step. Then, we apply contrast stretching, histogram equalization, and adaptive equalization to all the CT scans to reduce the noise ratio. Further, a pipeline is designed to effectively receive all the CT images and forward them to DenseNet201 for feature extraction. In fully connected layers, we use 3 + 9 dense layers. We obtain 88, 88, 89, and 90 percent accuracy, sensitivity, specificity, and AUC, respectively, on the test set. In the future, we aim to use gradient-weighted class activation mapping (GRAD-CAM, (explainable AI)) to visualize the decision of the CNN model in each scan.

## Figures and Tables

**Table 1:** Overview of state of the art.

Author	Model	Dataset Type	Dataset Repository	Results	Year
Tajbakhsh et al. [21]	CNN	CTPA	Private dataset + PE challenge	Sensitivity: 0.83	2015
X-Yang et al. [10]	CNN	CTPA	Private dataset + PE challenge (2019)	Sensitivity 0.75	2019
Weifang Liu et al. [22]	D-L-CNN	CTPA	Private dataset	AUC: 0.926, Sensitivity: 0.94, Specificity: 0.76	2020
Shih-Cheng Huang et al. [7]	PENet.77-layer 3D CNN model	CTPA imaging	Private dataset	AUC: 0.85, Sensitivity: 0.75, Specificity: 0.80, Accuracy: 0.78	2020
Thomas Weikert et al. [23]	Deep-CNN	CTPA	Private dataset	Sensitivity: 0.92, Specificity: 0.95	2020
Aditya Mohan et al. [24]	Xception-CNN	CTPA	RSNA-PE challenge-(2020)	Accuracy: 0.90	2020
Deepta Rajan et al. [25]	2D U-Net	CTs	Private dataset	AUC: 0.94	2020
Tuomas Vainio et al. [26]	CNN	CTPA	Private dataset	AUC: 0.87	2021
Nahid ul Islam et al. [8]	SeResNextSe, ResNext50, SeXception, DenseNet121, ResNet18, ResNet50	CTPA	Kaggle-RSNA-PE-Challenge (2020)	AUC: 0.88, AUC: 0.89, AUC: 0.88, AUC: 0.88, AUC: 0.87, AUC: 0.86	2021
Sudhir Suman et al. [27]	CNN-LSTM	CTPA	Kaggle-RSNA-PE-Challenge (2020)	AUC: 0.95	2021
Ryan Schmid et al. [28]	D-L-algorithm	CTs	Private dataset	AUC: 0.79 Specificity: 0.99. Specificity: 0.99	2021

**Table 2:** Classification performance on test set.

Accuracy (%)	Sensitivity (%)	Specificity (%)	AUC (%)
0.88 ± 2	0.88 ± 2	0.89 ± 2	0.90 ± 2

**Table 3:** Comparison with state of the art.

Authors	Model	Dataset	AUC
Weifang Liu et al. [22]	CNN	CTPA	0.85
Sheh Cheng Huang et al. [7]	PENet.77-layered 3D CNN model	CTPA	0.85
Tuomas Vainio et al. [26]	CNN	CTPA	0.87
Ryan Schmid et al. [28]	CNN	CTs	0.79
Nahid ul Islam et al. [8]	Multi-model CNN	CTs	0.89
Our Proposed framework	CNN based on DenseNet201	CTPA	0.90 ± 2

## Data Availability

The data presented in this study are available on request from the authors.
